# Influence of temperature on twitch potentiation following submaximal voluntary contractions in human plantar flexor muscles

**DOI:** 10.14814/phy2.15802

**Published:** 2023-08-24

**Authors:** Kazutaka Ota, Kazushige Sasaki

**Affiliations:** ^1^ Department of Life Sciences, Graduate School of Arts and Sciences The University of Tokyo Tokyo Japan

**Keywords:** cooling, electrical stimulation, electromyography, fast‐twitch fiber, heating

## Abstract

This study aimed to clarify the influence of temperature on post‐activation twitch potentiation, a possible indicator of fast‐twitch fiber activation during a preceding voluntary contraction. Ten healthy males immersed their left lower leg in water of different temperatures (cold: 0°C, neutral: 32–35°C, hot: ~43°C) for 20 min each. In each temperature condition, they performed submaximal (10%–50% of maximal voluntary contraction torque measured before water immersion) and maximal plantar flexions. Immediately after each voluntary contraction, twitch contractions were evoked with supramaximal stimulation of the posterior tibial nerve. The magnitude of twitch potentiation, defined as a percent increase in twitch torque following a voluntary contraction, increased with the intensity of the preceding voluntary contraction. The magnitude of twitch potentiation after the maximal voluntary contraction was smaller in Cold than in the other temperature conditions. However, temperature had no influence on the relative magnitude of twitch potentiation following the submaximal contractions. In addition, there was no difference in electromyographic activity between the gastrocnemius and soleus muscles in any temperature conditions. Collectively, the temperature dependence was not observed when using twitch potentiation or electromyographic amplitude as an indicator of fast‐twitch fiber activation during brief submaximal voluntary contractions.

## 1 INTRODUCTION

Temperature has a great influence on exercise performance by affecting muscle contractile properties and neuromuscular activity. For example, muscle cooling prolongs twitch contraction and relaxation time (Bigland‐Ritchie et al., 1992; Davies et al., 1982; Davies & Young, 1983; Mallette et al., 2018, 2019, 2021). In addition, muscle cooling has been reported to decrease peak twitch and maximal voluntary contraction (MVC) torque (Davies et al., 1982; Davies & Young, 1983; Gossen et al., 2001; Mallette et al., 2018, 2019, 2021). The decrease in MVC torque may be attributed to reduced force‐generating capacity because the recorded amplitude of surface electromyography (EMG) remains constant or even increases due to muscle cooling (Mallette et al., 2018, 2019, 2021; Thornley et al., 2003; Wakabayashi et al., 2017).

On the other hand, fewer studies have examined the effects of muscle heating on performance than those of muscle cooling. It has been reported that muscle heating shortens twitch contraction and relaxation time, while peak twitch and MVC torque remain unchanged (Davies et al., 1982; Davies & Young, 1983; Mallette et al., 2019, 2021; Mornas et al., 2021). The mechanisms underlying the asymmetric influence of temperature on muscle force production, especially during voluntary contractions, are not well understood.

Animal studies suggest that changes in muscle force‐generating capacity with temperature may depend on muscle fiber type. As muscle temperature decreases, twitch contraction force decreases in the slow‐twitch soleus muscle but increases in the fast‐twitch extensor and flexor digitorum longus muscles in small mammals (Buller et al., 1984; Close & Hoh, 1968). Therefore, at low muscle temperatures, fast‐twitch fibers have an advantage over slow‐twitch fibers in twitch contraction force. Furthermore, it was shown that cooling resulted in additional recruitment of fast‐twitch fibers in carp, likely compensating for the reduced force‐generating capacity of slow‐twitch fibers (Rome et al., 1984).

There is currently no method to noninvasively distinguish between the activities of slow‐ and fast‐twitch fibers of human muscle. However, we previously showed that the increase in twitch contractile force immediately after a voluntary contraction, called post‐activation twitch potentiation (TP), can be a measure of fast‐twitch fiber activation during the preceding contraction (Sasaki et al., 2012) if the contraction duration is short (Hamada et al., 2000; Vandervoort & McComas, 1983). A major mechanism of TP is considered an increase in Ca^2+^ sensitivity of the contractile proteins (Sweeney et al., 1993) due to the phosphorylation of myosin light chain (Zhi et al., 2005), which is much more pronounced in fast‐twitch fibers than in slow‐twitch fibers (Moore & Stull, 1984). In fact, the magnitude of TP has been used to account not only for differences in fast‐twitch fiber recruitment between voluntary and electrically evoked contractions (Regina Dias Da Silva et al., 2015; Requena et al., 2008) but also for additional recruitment of fast‐twitch fibers with increasing contraction intensity (Sasaki et al., 2012). While the magnitude of TP induced by high‐frequency tetanus has been shown to depend on temperature (Buller et al., 1984; Gossen et al., 2001; Malak et al., 2023; Manning & Stull, 1982; Moore et al., 1990), the magnitude of TP following a submaximal voluntary contraction can be used for clarifying the temperature‐dependent change in fast‐twitch fiber activation when normalized to the reference value (e.g., the magnitude of TP immediately after a few seconds of MVC). Therefore, this study aimed to explore the temperature dependence of the magnitude of TP immediately after submaximal voluntary contractions in human plantar flexor muscles. We hypothesized that the magnitude of TP after submaximal voluntary contractions increases with decreasing temperature.

## 2 METHODS

### 2.1 Participants

Ten healthy male volunteers participated in this study. Their mean (SD) age, height, and body mass were 25.7 (7.2) years, 178.7 (5.7) cm, and 78.6 (11.3) kg, respectively. Seven of them were regularly involved in vigorous physical activities such as resistance exercise, while the others occasionally engaged in recreational sports. A priori sample size calculation was performed with G*Power version 3.1.9.6. (Heinrich Heine Universität Düsseldorf) using a within‐participants analysis of variance (ANOVA) with a statistical power of 0.8 and an alpha error of 0.05. Seven participants were required to detect an effect size (Cohen's *f*) of 0.6, which was based on a previous report on the effect of muscle cooling and heating on peak twitch torque (Mallette et al., 2021). We decided to increase the number to 10 because we also measured other variables.

Before enrollment, written informed consent was obtained from each participant in accordance with the Declaration of Helsinki. This study was approved by the Ethical Review Committee for Experimental Research involving Human Subjects, Graduate School of Arts and Sciences and the College of Arts and Sciences, The University of Tokyo (Issue number: 885‐2).

### 2.2 Joint torque measurement

A custom‐designed ankle dynamometer was used to measure ankle joint torque produced by the plantar flexor and dorsiflexor muscles. The participant rested in a prone position with the left knee fully extended. The left foot was fixed at an ankle joint angle of 90° (neutral position) and attached firmly to a footplate installed in the dynamometer using inelastic straps. The footplate was positioned so that its rotational axis coincided with the anatomical axis of the ankle. The torque signal was obtained with a load cell (LUX‐B‐2KN‐ID, Kyowa Electronic Instruments) attached to the beam of the footplate and digitized at a sampling rate of 10 kHz by using a data acquisition system (PowerLab/16SP, ADInstruments).

### 2.3 Electromyography

EMG signals from the lateral gastrocnemius (LG), medial gastrocnemius (MG), soleus (Sol), and tibialis anterior (TA) muscles were obtained with a pair of Ag–AgCl surface electrodes (F‐150, Nihon Koden). Before the electrode placement, the skin was shaved, cleaned with alcohol, and abraded to reduce electrode impedance. The skin‐electrode impedance was confirmed to be low (<5 kΩ) with a digital tester (TDX‐200, Ohm Electric). The electrodes were placed on the mid‐belly of each muscle at an interelectrode distance of 25 mm. With regard to the electrode location, we followed the SENIAM (Surface Electromyography for the Non‐Invasive Assessment of Muscles) recommendations (Hermens et al., 2000) except for Sol, in which the electrodes were placed on the lateral part as opposed to the medial part recommended by the SENIAM. This is because the Sol electrodes placed medially tend to suffer from crosstalk (Bogey et al., 2000; Péter et al., 2019). The reference electrode was placed on the tibial belly. All EMG signals were amplified (gain 1000×) with an AC amplifier (AB‐610J, Nihon Koden), band‐pass filtered (5–1000 Hz), and sampled at 10 kHz by using the data acquisition system.

### 2.4 Electrical stimulation

Twitch contraction of plantar flexor muscles was evoked with a 0.5 ms rectangular pulse delivered by an electric stimulator (SEN‐3401, Nihon Koden) with a stimulus‐isolation unit (SS‐2185, Nihon Koden) through two Ag–AgCl surface electrodes (the same as used for EMG recordings). The cathode was placed on the posterior tibial nerve in the popliteal fossa, while the anode was placed on the anterior surface of the knee, just proximal to the patella. The voltage of the rectangular pulse was progressively increased until no further increase in twitch torque or M‐wave in triceps surae muscles was observed. The stimulus intensity was then increased by ~10% to ensure supramaximal stimulation and was maintained throughout the experiment. In an additional experiment on two participants, we found that the supramaximality of electrical stimulation was not compromised by the change in temperature (Figure S1), which is consistent with an earlier observation on the human triceps surae (Davies et al., 1982).

### 2.5 Muscle temperature measurement

The temperature in the plantar flexor muscles was noninvasively measured with a zero‐heat‐flow method. A temperature‐compensated probe (PD‐1, Terumo Corp) connected to an electronic thermometer (Coretemp CM‐210, Terumo Corp), with which the temperature 10 mm below the skin surface can be measured, was placed over the point approximately 1.5 cm proximal to the gastrocnemius muscle‐tendon junction. The temperature‐compensated probe works by preventing heat loss from the tissue below the probe by actively heating the tissue until no temperature gradient exists across the probe (Brajkovic & Ducharme, 2005). Although the muscle temperature measured with the zero‐heat‐flow method was not representative of the actual muscle temperature because the probe heated the muscle by ~2°C during its operation (Brajkovic & Ducharme, 2005), we used the method to noninvasively measure the differences in muscle temperature between the conditions.

### 2.6 Experimental procedure

**FIGURE 1:**
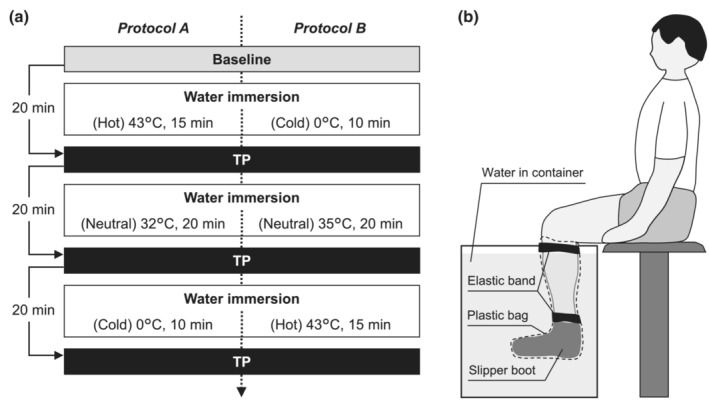
(a) Experimental protocols and (b) setup of water immersion. Ten participants were randomly assigned to either Protocol A or B by five each. In “Baseline” session, the participant performed maximal voluntary contractions following the determination of the supramaximal stimulus intensity. The participants immersed their left lower leg in water of different temperatures for about 20 min. After each water immersion, “TP (twitch potentiation)” sessions were completed, in which unpotentiated and potentiated twitches were recorded.

The present study used a within‐participants design, where all participants underwent three temperature conditions (Hot, Neutral, and Cold) in 1 day (Figure 1a) to ensure the same placement of EMG and stimulation electrodes throughout the experiment. They were instructed to refrain from strenuous exercise for 24 h before the experiment.

After skin preparation and electrode placement were carried out, each participant underwent one “Baseline” session and then three “TP” sessions with different muscle temperatures (Figure 1a). Manipulation of muscle temperature by water immersion (see below) was performed between the sessions.

In Baseline session, the supramaximal stimulus intensity was determined first. Next, the participant performed three 6‐s isometric MVC (MVC_pre_) of plantar flexor muscles after several warm‐up contractions. During MVC, the participant received verbal encouragement and visual feedback of the torque signal provided by data‐recording software (LabChart 7, AD Instrument). At least 1 min of rest was allowed between contractions. Two 6‐s isometric MVC of dorsiflexor muscles were performed in the same way to normalize TA activity during the plantar flexion (antagonistic co‐contraction) with its maximal activity.

Ten participants were randomly assigned to two protocols, different in the order of temperature conditions (Figure 1a). Muscle temperature was altered by immersing the left lower leg in a large container filled with water of different temperatures for about 20 min each in a sitting position (Figure 1b). Specifically, water temperature of ~43°C was used for Hot, 32–35°C for Neutral, and 0°C (ice water) for Cold conditions. The water temperature was constantly checked with a thermometer (Multi‐thermometer, Japan Pet Design) and adjusted to a target level by adding hot or cold water to the container. The left foot was covered with a slipper made of brushed material to reduce pain and discomfort. The lower leg was covered with a plastic bag to prevent the skin and electrodes from getting wet. A hot pack (Far‐infrared Cosmopackfit, NihonEnseki) and a cold pack (Polar Care Kodiak, Breg) were wrapped around the left lower leg during TP sessions in Hot and Cold to maintain muscle temperature achieved with the water immersion, respectively.

In each TP session, first, three supramaximal twitches with a 3‐s interval were recorded as an unpotentiated baseline. Afterward, a series of 6‐s submaximal voluntary contractions arranged in ascending order (Sasaki et al., 2012) of contraction intensity (from 10% to 50% of MVC_pre_ at 10% intervals) was performed. TP in the human plantar flexor muscles was typically observed at contraction intensities above 30%–40% of MVC (Sasaki et al., 2012). To test the possible effect of temperature on the threshold intensity at which TP becomes measurable, the intensity of submaximal contractions was set from 10% to 50% of MVC_pre_. We excluded higher intensities to ensure that all participants could complete a series of contractions with negligible fatigue even if the force‐generating capacity changes with temperature, as suggested previously (Cornwall, 1994; Davies et al., 1982; Davies & Young, 1983). During the submaximal voluntary contraction, the participant was instructed to match the torque trace gradually with a reference line displayed on a screen. Each contraction was immediately (within 3 s) followed by three supramaximal twitches with a 3‐s interval and at least a 1‐min rest. Finally, a single 6‐s MVC (MVC_post_) followed by three supramaximal twitches was performed.

### 2.7 Data analysis

Muscle temperature was expressed by the average obtained at the start and end of each TP session. The torque signal was digitally low pass filtered (zero‐lag, fourth‐order Butterworth filter) with a cutoff frequency of 25 Hz (Albert et al., 2006; McCrory et al., 2009). The following parameters of twitch contraction were calculated: the highest value of twitch torque (peak torque), the time from electrical stimulus to the onset of torque production (electromechanical delay), the time from electrical stimulus to peak twitch torque (time to peak torque), and the time for peak twitch torque to decay by 50% (half relaxation time). In addition, root‐mean‐square amplitude (RMS) of M‐wave was calculated during a fixed 30‐ms epoch starting 5 ms after delivery of the electrical stimulus (Cronin et al., 2015) for LG, MG, and Sol. Data obtained from three consecutive twitches were averaged. The magnitude of TP was defined as a percent increase in twitch torque following a voluntary contraction, expressed as a percentage. Because the magnitude of TP itself has a temperature dependence (Buller et al., 1984; Gossen et al., 2001; Malak et al., 2023; Manning & Stull, 1982; Moore et al., 1990), we also calculated the magnitude of TP relative to the maximal value, typically observed immediately after MVC_post_. For voluntary contractions, the average torque and the RMS of EMG in LG, MG, Sol, and TA were calculated for 3 s. The calculation period was automatically determined such that the average torque was maximized in MVC (MVC_pre_ and MVC_post_) and that the torque variability (i.e., the standard deviation divided by the average) was minimized in submaximal contractions. RMS of EMG in LG, MG, and Sol was normalized to that of maximal M‐wave evoked immediately after each voluntary contraction (Arabadzhiev et al., 2010; Racinais, 2013) to account for the changes in the recorded waveform of motor unit action potential with temperature (Dewhurst et al., 2005; Racinais, 2013) and by the preceding voluntary contractions (Hamada et al., 2000; Sasaki et al., 2012). Except for TA, we did not express the submaximal EMG as a percentage of MVC, considering a possible change in maximal voluntary activation with temperature.

### 2.8 Statistics

Data are expressed as mean and SD. The differences in means across the three temperature conditions (e.g., muscle temperature, twitch contractile properties, and MVC_post_ torque) were analyzed with a one‐way analysis of variance (ANOVA) with repeated measures. For the magnitude of TP and M‐wave amplitude after voluntary contractions, a two‐way (temperature condition and contraction intensity) ANOVA with repeated measures was used to test whether the effect of contraction intensity was different between temperature conditions. The two‐way (temperature condition and muscle) ANOVA with repeated measures was also used for the EMG amplitude in plantar flexors during MVC_post_. A three‐way ANOVA with repeated measures was used to test main effects and interactions of muscle, temperature condition and contraction intensity (within‐participants factors) on EMG amplitude during submaximal voluntary contractions. Because the participants performed well on the torque‐matching task (see Section 3), we treated the target torque level as an independent variable in the ANOVA model. Where appropriate, ANOVA was followed by post hoc analysis, Student's paired *t*‐test with the Shaffer procedure. Linear regression analysis was used to assess the influence of individual differences in muscle temperature on the magnitude of submaximal TP averaged over five contraction intensities. To eliminate the effect of contraction intensity, the magnitude of submaximal TP was standardized before averaging, to a mean of zero and a SD of one in each contraction intensity. In all statistical tests, *p* < 0.050 was considered significant. As a measure of effect size for ANOVA, post hoc paired *t*‐test, and linear regression analysis, we calculated generalized *η*‐squared (*η* ^2^), Cohen's *d*, and *r*‐squared (*r* ^2^), respectively. The analyses were carried out using R version 4.2.2 (R Foundation for Statistical Computing) and the R function “anovakun” version 4.8.6 (Iseki, 2023).

## 3 RESULTS

### 3.1 Muscle temperature

**FIGURE 2:**
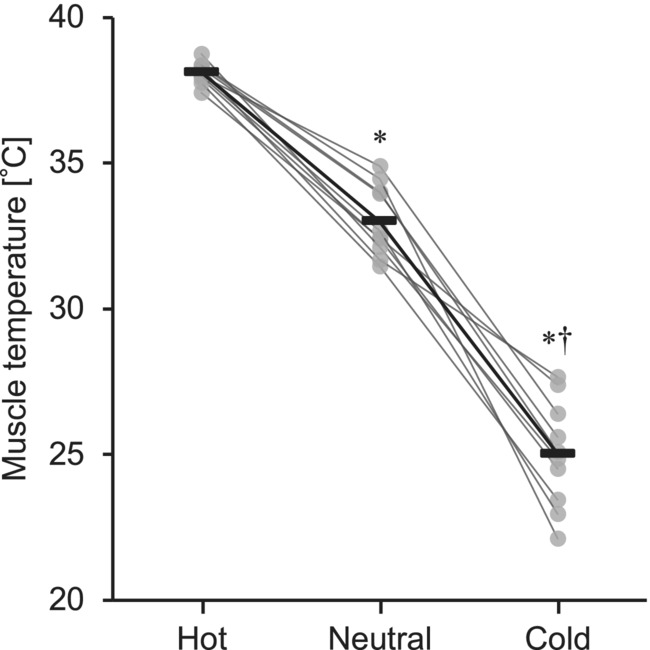
Temperature in the triceps surae muscles. Individual and average values are presented as gray circles and black bars, respectively. *Significant difference from Hot, *p* < 0.001. ^†^Significant difference from Neutral, *p* < 0.001. Obtained from ANOVA with repeated measures and post hoc Student's paired *t*‐test with the Shaffer procedure.

Figure 2 shows differences in the noninvasively measured temperature in the triceps surae muscles. The one‐way ANOVA revealed a significant main effect of temperature condition (*η* ^2^ = 0.951, *p* < 0.001). The muscle temperature was higher in Hot (38.1 ± 0.4°C, *d* = 5.649, *p* < 0.001) and lower in Cold (25.0 ± 1.8°C, *d* = 5.130, *p* < 0.001) than in Neutral (33.0 ± 1.2°C). The within‐session temperature variances (calculated as the absolute difference between the start and the end of TP session) were as follows: 0.48 ± 0.41°C in Hot, 0.40 ± 0.37°C in Neutral, and 2.20 ± 1.10°C in Cold.

### 3.2 Unpotentiated twitch torque and M‐wave amplitude

**FIGURE 3:**
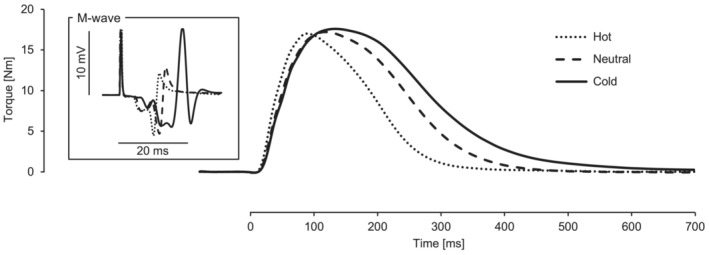
Typical recordings of unpotentiated twitch torque and M‐wave in medial gastrocnemius muscle.

Figure 3 shows typical recordings of unpotentiated twitch torque and M‐wave in the MG, illustrating the temperature dependence of twitch contraction time and M‐wave amplitude, but not of peak twitch torque. Table 1 summarizes twitch contractile properties and M‐wave amplitude in unpotentiated muscles in each temperature condition. The one‐way ANOVA revealed a significant main effect of temperature condition on electromechanical delay, time to peak torque, and half relaxation time, but not on peak torque. The post hoc analysis revealed that these measures were consistently shorter in Hot (*d* ≥ 0.707, *p* ≤ 0.001) and longer in Cold (*d* ≥ 0.573, *p* < 0.001) than in Neutral. There was a significant main effect of temperature condition on M‐wave amplitude in MG and Sol, but not in LG. M‐wave amplitude in MG was larger in Cold than in Neutral and Hot (*d* ≥ 1.031, *p* < 0.001 vs. both). M‐wave amplitude in Sol was smaller in Hot (*d* = 0.425, *p* = 0.021) and larger in Cold (*d* = 0.442, *p* = 0.042) than in Neutral.

**TABLE 1:** Twitch contractile properties and root‐mean‐square amplitude of M‐wave in unpotentiated muscles.

	Hot	Neutral	Cold	ANOVA	
				*η* ^2^ value	*p* value
Peak torque (N m)	13.9 ± 3.7	13.1 ± 3.0	13.2 ± 2.9	0.014	0.174
EMD (ms)	12.4 ± 1.6	13.5 ± 1.5^a^	14.4 ± 1.6^a^ ^,^ ^b^	0.231	<0.001
TPT (ms)	97.5 ± 10.4	116.1 ± 12.5^a^	141.5 ± 13.3^a^ ^,^ ^b^	0.710	<0.001
HRT (ms)	84.3 ± 14.8	104.9 ± 22.4^a^	146.3 ± 15.1^a^ ^,^ ^b^	0.700	<0.001
LG (mV)	0.625 ± 0.498	0.632 ± 0.491	0.636 ± 0.503	<0.001	0.903
MG (mV)	0.877 ± 0.640	0.904 ± 0.569	1.615 ± 0.875^a^ ^,^ ^b^	0.224	<0.001
Sol (mV)	0.409 ± 0.217	0.502 ± 0.220^a^	0.623 ± 0.318^a^ ^,^ ^b^	0.115	0.002

*Note*: Values are mean and SD (*n* = 10).

Abbreviations: EMD, electromechanical delay; HRT, half relaxation time; LG, lateral gastrocnemius; MG, medial gastrocnemius; Sol, soleus; TPT, time to peak torque.

^a^ Significant difference from Hot, *p* < 0.050.

^b^ Significant difference from Neutral, *p* < 0.050. Obtained from ANOVA with repeated measures and post hoc Student's paired *t*‐test with the Shaffer procedure.

### 3.3 Maximal voluntary contraction torque and electromyographic activity

Table 2 summarizes torque and EMG amplitude during MVC_post_ in each temperature condition. The one‐way ANOVA revealed no significant main effect of temperature condition on the torque or co‐contraction level of TA. EMG amplitude in plantar flexors was affected by the temperature. The two‐way ANOVA revealed a significant main effect of temperature condition (*η* ^2^ = 0.052, *p* = 0.026), but not a significant main effect of muscle (*η* ^2^ = 0.078, *p* = 0.121), or interaction of temperature condition by muscle (*η* ^2^ = 0.020, *p* = 0.261). EMG amplitude was significantly smaller in Hot than in the other temperature conditions (*d* ≥ 0.218, *p* = 0.035 vs. both).

**TABLE 2:** Maximal voluntary contraction torque and root‐mean‐square amplitude of electromyographic signals.

	Hot	Neutral	Cold	ANOVA	
				*η* ^2^ value	*p* value
Torque (N m)	95.3 ± 17.2	94.9 ± 16.7	92.2 ± 15.2	0.008	0.443
LG (%M‐wave)	13.2 ± 4.5	13.4 ± 4.6	15.2 ± 4.9	‐	‐
MG (%M‐wave)	14.4 ± 6.5	17.6 ± 8.2	17.5 ± 5.8	‐	‐
Sol (%M‐wave)	11.1 ± 4.3	11.3 ± 3.8	15.4 ± 8.4	‐	‐
LG + MG + Sol (%M‐wave)	12.9 ± 5.2	14.1 ± 6.3^a^	16.0 ± 6.4^a^	0.052	0.026
TA (%MVC_pre_)	15.1 ± 7.2	16.3 ± 9.5	18.9 ± 11.1	0.031	0.125

*Note*: Values are mean and SD (*n* = 10). A “hyphen” (‐) indicates that a grade is not available.

Abbreviations: LG, lateral gastrocnemius; MG, medial gastrocnemius; MVC_pre_, maximal voluntary contraction of dorsiflexor muscles performed at baseline; Sol, soleus; LG + MG + Sol, averaged data of LG, MG, and Sol; TA, tibialis anterior.

^a^ Significant difference from Hot, *p* < 0.050. Obtained from ANOVA with repeated measures and post hoc Student's paired *t*‐test with the Shaffer procedure.

### 3.4 Submaximal voluntary contraction torque and electromyographic activity

Participants correctly completed torque‐matching tasks during submaximal voluntary contractions. The deviations of the torque from the target torque were quite small: less than 5% in 140 of the 150 torque‐matching tasks (overall mean and SD: 2.1 ± 2.1%).

**FIGURE 4:**
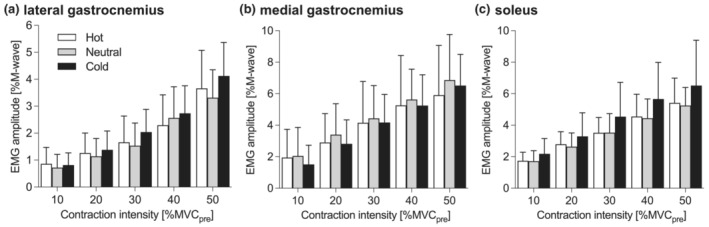
Root‐mean‐square amplitude of electromyographic (EMG) signals from (a) lateral gastrocnemius, (b) medial gastrocnemius, and (c) soleus. MVC_pre_, maximal voluntary contraction torque determined at baseline. Values are mean and SD (*n* = 10).

For EMG amplitude during the submaximal voluntary contractions (Figure 4), the three‐way ANOVA revealed a significant main effect of muscle (*η* ^2^ = 0.280, *p* = 0.015), main effect of contraction intensity (*η* ^2^ = 0.429, *p* < 0.001), interaction of muscle by contraction intensity (*η* ^2^ = 0.033, *p* = 0.001), and interaction of temperature condition by contraction intensity (*η* ^2^ = 0.005, *p* = 0.035), but not a significant main effect of temperature condition (*η* ^2^ = 0.011, *p* = 0.138), interaction of muscle by temperature condition (*η* ^2^ = 0.022, *p* = 0.219), or three‐way interaction (*η* ^2^ = 0.003, *p* = 0.646). While a post hoc analysis did not detect a significant difference between the temperature conditions, EMG amplitude was smaller in LG than in the other muscles (*d* = 1.030, *p* = 0.004 vs. MG; *d* = 1.067, *p* = 0.006 vs. Sol).

The co‐contraction level of TA during submaximal plantar flexions was also affected only by contraction intensity. There was a significant main effect of contraction intensity (*η* ^2^ = 0.243, *p* = 0.002), but not a significant main effect of temperature condition (*η* ^2^ = 0.015, *p* = 0.141), or interaction (*η* ^2^ = 0.004, *p* = 0.320).

### 3.5 Twitch torque and M‐wave amplitude after voluntary contraction

**FIGURE 5:**
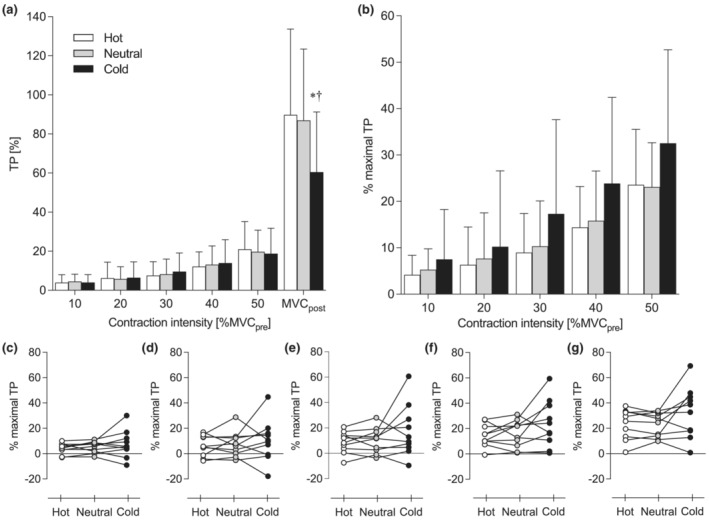
(a) The magnitude of post‐activation twitch potentiation (TP). (b) The magnitude of submaximal TP relative to the maximal TP, that is, TP determined immediately after a maximal voluntary contraction performed at the end of each condition (MVC_post_). (c–g) Individual data in 10%, 20%, 30%, 40%, and 50% MVC_pre_ are also presented, respectively. MVC_pre_, maximal voluntary contraction torque determined at baseline. Values are mean and SD (*n* = 10). *Significant difference from Hot, *p* < 0.010. ^†^Significant difference from Neutral, *p* < 0.001. Obtained from ANOVA with repeated measures and post hoc Student's paired *t*‐test with the Shaffer procedure.

Figure 5 shows the differences in the magnitude of post‐activation twitch potentiation (TP) following submaximal and maximal voluntary contractions. The magnitude of TP was affected by both contraction intensity and temperature (Figure 5a). The two‐way ANOVA revealed a significant interaction of temperature condition by contraction intensity (*η* ^2^ = 0.086, *p* < 0.001), and main effect of contraction intensity (*η* ^2^ = 0.734, *p* < 0.001), but not a significant main effect of temperature condition (*η* ^2^ = 0.013, *p* = 0.061). The post hoc analysis revealed that the magnitude of TP increased with the intensity of the preceding voluntary contraction and was maximized after MVC_post_ in each temperature condition. The maximal magnitude of TP observed after MVC_post_ was significantly smaller in Cold than in the other temperature conditions (*d* = 0.783, *p* < 0.001 vs. Neutral; *d* = 0.771, *p* = 0.007 vs. Hot). When the magnitude of TP was normalized with that after MVC_post_ (Figure 5b), the two‐way ANOVA revealed a significant main effect of contraction intensity (*η* ^2^ = 0.386, *p* < 0.001), but not a significant main effect of temperature condition (*η* ^2^ = 0.051, *p* = 0.185) or interaction (*η* ^2^ = 0.023, *p* = 0.219). Although the temperature did not affect the magnitude of TP following submaximal contractions, there were individual differences, particularly in Cold (Figure 5c–g). The individual difference in muscle temperature did not affect the relative magnitude of submaximal TP in Hot (*r* ^2^ = 0.228, *n* = 10, *p* = 0.163), Neutral (*r* ^2^ = 0.008, *n* = 10, *p* = 0.812), or Cold (*r* ^2^ = 0.052, *n* = 10, *p* = 0.525).

M‐wave amplitude in LG increased with the intensity of the preceding contraction. The two‐way ANOVA revealed a significant main effect of contraction intensity on LG (*η* ^2^ = 0.100, *p* = 0.017), but not on MG (*η* ^2^ = 0.032, *p* = 0.221) or Sol (*η* ^2^ = 0.059, *p* = 0.071). The two‐way ANOVA did not reveal a significant main effect of temperature condition (LG, *η* ^2^ = 0.034, *p* = 0.861; MG, *η* ^2^ = 0.020, *p* = 0.540; Sol, *η* ^2^ = 0.075, *p* = 0.198) or interaction (LG, *η* ^2^ = 0.022, *p* = 0.292; MG, *η* ^2^ = 0.010, *p* = 0.716; Sol, *η* ^2^ = 0.061, *p* = 0.062).

## 4 DISCUSSION

The aim of the present study was to clarify the temperature dependence of the magnitude of post‐activation twitch potentiation (TP). We hypothesized that the magnitude of TP immediately after submaximal contractions changes with temperature. The major finding is that the temperature effects were evident in the twitch time properties, but not in the magnitude of TP after submaximal voluntary contractions.

There have been inconsistent observations on the temperature dependence of neuromuscular activity during submaximal voluntary contraction. Some studies reported an increase in surface EMG activity with muscle cooling (Mallette et al., 2019, 2021; Wakabayashi et al., 2017), while others reported a decrease (Petrofsky & Lind, 1980) or no change (Mallette et al., 2018; Thornley et al., 2003). While surface EMG signals represent the temporal and spatial summation of motor unit action potentials, recent technical development allows us to decompose them into individual motor unit potentials (Mallette et al., 2018, 2021). However, the action potential amplitude in an individual motor unit estimated with the decomposition may not depend on the fiber type. Hence, it remains unclear whether muscle fiber activation has temperature dependence. In contrast to surface EMG activity, TP has been found to be fiber‐type dependent in cats (Bagust et al., 1974), rabbits (Moore et al., 1985), and rats (Malak et al., 2023; Manning & Stull, 1982; Moore & Stull, 1984). The association of TP with muscle fiber type has been shown in human studies by comparison of muscles with different fiber‐type compositions (Vandervoort & McComas, 1983) and of individuals with a predominance of fast‐ and slow‐twitch fibers (Hamada et al., 2000, 2003). Therefore, we attempted to investigate the temperature dependence of the fast‐twitch fiber activation by assessing TP (Sasaki et al., 2012) in addition to EMG.

No temperature effect was found on the relative magnitude of TP (Figure 5b), indicative of fast‐twitch fiber activation during a preceding voluntary contraction (Sasaki et al., 2012). We did not find a difference in EMG activity between gastrocnemius muscles (LG and MG) with a “mixed” fiber‐type composition and Sol characterized by the predominance of slow‐twitch fibers (Johnson et al., 1973) in any temperature conditions. These results suggest that fast‐twitch fiber activation of human plantar flexors during submaximal voluntary contractions is insensitive to temperature change. Previous studies have demonstrated the possibility that cooling induces additional recruitment of fast‐twitch fibers or high‐threshold motor units to compensate for the reduction in motor performance (Fujimoto et al., 2016; Mallette et al., 2018; Rome et al., 1984; Wakabayashi et al., 2018). In the present study, however, there should be no need for a compensatory increase in fast‐twitch fiber activation because the force‐generating capacity of plantar flexors, represented by the unpotentiated peak twitch torque (Table 1) and MVC_post_ torque (Table 2), did not change with the temperature.

One possible reason why MVC torque was not influenced by temperature is that temperature changes altered EMG activity (Table 2). In fact, we found that EMG activity during MVC_post_ is smaller in Hot than in the other temperature conditions regardless of muscle, which is consistent with a previous observation on the flexor carpi radialis (Mallette et al., 2019). The temperature effect on EMG activity during MVC_post_ may be accounted for by several factors including not only the changes in muscle contractile properties but also the changes in muscle activation properties coming from a wide variety of inputs.

It is unlikely that negligible temperature dependence of the magnitude of TP was due to an insufficient change in muscle temperature. We included not only cold but also hot conditions to test the temperature effect over a wide range. Although limited data are available in humans, the changes in muscle temperature achieved with water immersion in this study (~13°C decrease in Cold and ~5°C increase in Hot) were larger than those in studies examining human neuromuscular activity (~8°C decrease and ~3°C increase due to cold and hot water immersion, respectively) (Davies et al., 1982; Davies & Young, 1983). Further, we found that muscle cooling increased and heating decreased the electromechanical delay and the time course of twitch contractions (Table 1), which agrees with many studies (Bigland‐Ritchie et al., 1992; Cè et al., 2013; Davies et al., 1982; Davies & Young, 1983; Mallette et al., 2018, 2019, 2021; Mornas et al., 2021; Racinais et al., 2017). In addition, individual differences in the relative magnitude of TP were not associated with those in muscle temperature in any temperature condition (*r* ^2^: 0.008–0.228).

Irrespective of temperature, the magnitude of TP increased with the intensity of the preceding voluntary contraction and was maximized after MVC_post_ (Figure 5a). The compound muscle action potential of LG also increased with the contraction intensity, which can be assumed to have a minor contribution to TP (Sasaki et al., 2012). Regarding the temperature dependence, the maximal TP, that is, TP determined immediately after MVC_post_, was significantly smaller in Cold than in the other temperature conditions (Figure 5a), which generally agrees with earlier observations on animal and human muscles (Buller et al., 1984; Gossen et al., 2001; Malak et al., 2023; Manning & Stull, 1982; Moore et al., 1990). Because the torque and EMG amplitude in MVC_post_ were not smaller in Cold than in the other conditions (Table 2), the suppression of the maximal TP in Cold would not reflect a decrease in neuromuscular activity. Rather, it may be due to limited room for improvement in twitch response at low muscle temperatures because the sensitivity of contractile proteins to Ca^2+^ in resting fast‐twitch fibers of rats was shown to increase as temperature decreased (Stephenson & Williams, 1985). In addition, Ca^2+^ release from the sarcoplasmic reticulum was reported to be suppressed at low temperatures in mouse muscle (Barclay, 2012). It should be emphasized, however, that the temperature dependence of the maximal TP would not invalidate the idea that the magnitude of TP immediately after a submaximal contraction can be a measure of fast‐twitch fiber activation during the preceding contraction if the magnitude of “submaximal TP” is normalized to that of maximal TP. Moreover, TP‐related alteration in motor unit firing rate during submaximal voluntary contractions, reported in the triceps brachii (Klein et al., 2001) and the tibialis anterior (Inglis et al., 2011), was assumed to be minimal in this study because of the relatively small magnitude of TP in plantar flexors as well as the ascending order of contraction intensities. In fact, the temperature did not affect the magnitude of TP except the one after MVC_post_ (Figure 5a).

Some limitations of this study should be acknowledged. First, the validity of TP as an indicator of fast‐twitch fiber activation has yet to be tested rigorously. The intensity dependence of TP even during low‐intensity voluntary contractions (Figure 5a) is consistent with our earlier observation (Sasaki et al., 2012) but somewhat contradictory to the predominance of slow‐twitch fibers in human plantar flexors, especially in the soleus (Johnson et al., 1973). In addition, TP is not established to be fiber‐type specific in human muscles, although the ability of TP is associated with fiber‐type composition (Hamada et al., 2000, 2003; Vandervoort & McComas, 1983). The temperature insensitivity of muscle fiber activation in human plantar flexors should be validated using different methods such as high‐density EMG (Mallette et al., 2018, 2021). Second, we used a zero‐heat‐flow method to noninvasively measure muscle temperature as with some previous studies (Kubo et al., 2011; Muraoka et al., 2005, 2008). The thermometer we used can measure the temperature 10 mm below the skin surface, whereas subcutaneous fat thickness on a posterior lower leg is ~6 mm in young males (Yagasaki & Toyokawa, 1989). Hence, the temperature we measured would reflect the temperature of the superficial part of plantar flexor muscles. Third, we did not assess the voluntary activation level during MVC_post_ using the interpolated twitch technique (Allen et al., 1995), despite the use of electrical stimulation. We assumed that muscle activity during MVC_post_ is sensitive to the core but not local temperature (Racinais, 2013; Thomas et al., 2006), but found that EMG activity during MVC_post_ was temperature dependent. Therefore, future studies should consider the possible change in maximal voluntary activation with temperature. Fourth, the triceps surae muscles are complex in terms of fiber‐type composition and anatomical configuration. Sensitivity to cooling and heating potentially differs between the gastrocnemius muscles and Sol, although we found no difference in EMG activity between the muscles in any temperature conditions. Finally, the present study examined only isometric contractions, which are typically used to study the temperature dependence of force‐generating capacity and neuromuscular activity (Bigland‐Ritchie et al., 1992; Davies et al., 1982; Davies & Young, 1983; Gossen et al., 2001; Mallette et al., 2018, 2019, 2021; Thornley et al., 2003; Wakabayashi et al., 2017). As the temperature dependence is known to be more pronounced in the contractile velocity and the rate of force development than in maximal force (Ranatunga, 1982), further research should include not only isometric contractions with a constant force but also dynamic contraction or ballistic force production (Coletta et al., 2018).

## 5 CONCLUSION

Immersing a lower leg in water with different temperatures resulted in significant changes in muscle temperature and the magnitude of twitch potentiation immediately after MVC. However, no temperature dependence was found on the magnitude of twitch potentiation after submaximal voluntary contractions. In addition, there was no difference in EMG activity between the gastrocnemius (mixed‐type) and soleus (slow‐type) muscles in any temperature conditions. Collectively, the temperature dependence was not observed when using twitch potentiation or EMG amplitude as an indicator of fast‐twitch fiber activation during brief submaximal voluntary contractions. Our findings are useful in exploring the temperature dependence of neuromuscular activity during voluntary contractions, although further studies are needed to generalize the findings to other muscle groups and contraction types.

## AUTHOR CONTRIBUTIONS

Conceptualization and methodology: Kazutaka Ota, Kazushige Sasaki; data curation, formal analysis and investigation: Kazutaka Ota; software: Kazutaka Ota, Kazushige Sasaki; writing—original draft preparation: Kazutaka Ota; writing—review and editing: Kazushige Sasaki; visualization: Kazutaka Ota; funding acquisition and supervision: Kazushige Sasaki.

## FUNDING INFORMATION

This work was supported by Grants‐in‐Aid for Scientific Research (KAKENHI) from Japan Society for the Promotion of Science to K.S. (Grant Numbers JP19H01085 and JP22K11524).

## CONFLICT OF INTEREST STATEMENT

No conflict of interest, financial, or otherwise, are declared by the authors.

## ETHICS STATEMENT

This study was performed in line with the principles of the Declaration of Helsinki. Approval was granted by the Ethical Review Committee for Experimental Research involving Human Subjects, Graduate School of Arts and Sciences and the College of Arts and Sciences, The University of Tokyo (Issue number: 885‐2). Written informed consent was obtained from the participants.

## Supporting information


Figure S1.

## Data Availability

The datasets generated and/or analyzed during the current study are available from the corresponding author on reasonable request.
