# Supplementary material for: Influence of temperature on twitch potentiation following submaximal voluntary contractions in human plantar flexor muscles
Source: Physiol Rep. 2023 Aug 24;11(16):e15802. doi: 10.14814/phy2.15802 (PMC10449604; doi:10.14814/phy2.15802)
Supplement: Supplementary file 1 — Figure S1. [file PHY2-11-e15802-s001.docx]

**Figure S1**. Relationship between stimulus intensity and twitch torque (i.e., twitch recruitment curve) determined before (Baseline) and after immersion in hot (Hot), tepid (Neutral), and cold (Cold) water. Data from participants A (left) and B (right) were fitted to a Boltzmann function. In all conditions, twitch torques reached a plateau at supramaximal intensity (vertical dotted line).
